# Mitochondrial and Neuronal Dysfunctions in L1 Mutant Mice

**DOI:** 10.3390/ijms23084337

**Published:** 2022-04-14

**Authors:** Ludovica Congiu, Viviana Granato, Gabriele Loers, Ralf Kleene, Melitta Schachner

**Affiliations:** 1 Zentrum für Molekulare Neurobiologie, Universitätsklinikum Hamburg-Eppendorf, Falkenried 94, 20251 Hamburg, Germany; ludovica.congiu@zmnh.uni-hamburg.de (L.C.); viviana.granato@zmnh.uni-hamburg.de (V.G.); gabriele.loers@zmnh.uni-hamburg.de (G.L.); ralf.kleene@zmnh.uni-hamburg.de (R.K.); 2 Keck Center for Collaborative Neuroscience, Department of Cell Biology and Neuroscience, Rutgers University, Piscataway, NJ 08554, USA

**Keywords:** cell adhesion molecule L1, L1CAM, mitochondria, Complex I activity, ATP, neuronal survival, neurite outgrowth

## Abstract

Adhesion molecules regulate cell proliferation, migration, survival, neuritogenesis, synapse formation and synaptic plasticity during the nervous system’s development and in the adult. Among such molecules, the neural cell adhesion molecule L1 contributes to these functions during development, and in synapse formation, synaptic plasticity and regeneration after trauma. Proteolytic cleavage of L1 by different proteases is essential for these functions. A proteolytic fragment of 70 kDa (abbreviated L1-70) comprising part of the extracellular domain and the transmembrane and intracellular domains was shown to interact with mitochondrial proteins and is suggested to be involved in mitochondrial functions. To further determine the role of L1-70 in mitochondria, we generated two lines of gene-edited mice expressing full-length L1, but no or only low levels of L1-70. We showed that in the absence of L1-70, mitochondria in cultured cerebellar neurons move more retrogradely and exhibit reduced mitochondrial membrane potential, impaired Complex I activity and lower ATP levels compared to wild-type littermates. Neither neuronal migration, neuronal survival nor neuritogenesis in these mutants were stimulated with a function-triggering L1 antibody or with small agonistic L1 mimetics. These results suggest that L1-70 is important for mitochondrial homeostasis and that its absence contributes to the L1 syndrome phenotypes.

## 1. Introduction

Complex neural functions are achieved by the structural and functional interactions of neural activities, which strongly depend on the functional integrity of the mitochondria [1,2,3,4]. Mitochondria are maternally inherited cytoplasmic organelles which are considered to be the “powerhouse” of the cell, generating ATP for most, if not all, cellular activities [2,4,5,6,7]. Mitochondria play key roles in cellular homeostasis, especially in energy metabolism, redox system activity, elimination of reactive oxygen species (ROS), calcium signaling, senescence and apoptosis. Mitochondria are essential for nervous system functions, given the high energy demand of the neurons. Consequently, diseases affecting the mitochondrial oxidative phosphorylation system commonly exhibit neurological impairments [8,9,10]. Mitochondrial dysfunction contributes to the cognitive decline associated with neurodegenerative diseases [1,11,12,13]. However, mitochondria are a major source of function-adverse free radicals that underlie oxidative and nitrosative damage, which leads to a multitude of neurodegenerative diseases [3,11,14,15,16].

Mitochondria are not only important for the postmitotic and differentiated neurons, but also for neural progenitor cells during neurogenesis [17]. Thus, during brain development and in the adult nervous system, maintenance of mitochondrial integrity and homeostasis is extremely critical, being achieved through continual fusion, fission and mitophagy. Mitochondrial fusion allows the transfer of gene products between the mitochondria to assure optimal functioning, especially under metabolic and environmental stress. On the other hand, fission is crucial for mitochondrial division and quality control [18]. Dysfunctional mitochondria are eliminated by mitophagy, a selective autophagic process [19]. Impaired mitophagy and mitochondrial transport in axons correlate with neurodegenerative diseases [20]. An important parameter that is essential for determining cellular well-being is the mitochondrial membrane’s potential, as it is a direct measure of the cell’s ATP generating capability [21].

Because of the need to maintain and even improve mitochondrial function, investigations into mitochondrial heath are essential. A molecule that was recently shown to improve mitochondrial function is the cell adhesion molecule L1, a member of the immunoglobulin superfamily [22,23]. L1 contributes to neuronal migration and survival, axon outgrowth and fasciculation, synaptic plasticity and regeneration after trauma. Mutations of L1 in humans lead to the L1 syndrome, which is associated with neural malformations, such as hydrocephalus, corpus callosum agenesis, abnormalities of the corticospinal tract and severe disruptions of the enteric nervous system [24,25,26,27]. These histological features are associated with mental retardation, aphasia, spastic paraplegia, shuffling gait and adducted thumbs [24,25,26]. Genetic ablation (i.e., knock-out of *L1* in mice), inhibition of the mitochondrial import of L1-70 or prevention of myelin basic protein-mediated proteolytic generation of L1-70 in cell cultures all lead to reduced mitochondrial Complex I activity, impaired mitochondrial membrane potential, fusion, fission and motility, as well as increased retrograde mitochondrial transport [28]. Furthermore, adenine nucleotide translocator isoforms 1 and 2 at the plasma membrane that transport ATP were identified as L1 interaction partners [29]. However, how fragments or mutations of L1 influence mitochondrial activity and function has remained unknown. To address this question, we generated gene-edited mice lacking L1-70 (L1/687 and L1/858-863 mice [30]) and gene-edited mice with an aspartic acid to asparagine exchange at position 201 (D201N) and an L1 syndrome-like phenotype (L1-201 mice [31]) showing normal L1 proteolytic processing and all L1 fragments, including L1-70, but neural malformations and motor deficits [31] (unpublished data) (see also Supplementary Figure S1). Here, we report that neurons from L1-70-deficient mice display impaired L1-mediated neurite outgrowth, neuronal migration and neuronal survival. While L1 syndrome model mice show higher mitochondrial membrane potential, normal Complex I activity and ATP levels, as well as enhanced retrograde mitochondrial transport and higher levels of reactive oxygen species, mitochondria from L1-70 deficient mice exhibit lower membrane potential, lower ATP levels, enhanced retrograde transport and reduced Complex I activity compared to mitochondria from their wild-type littermates.

## 2. Results

### 2.1. Survival and Migration of Neurons and Neurite Outgrowth in L1/858-863 and L1/687 Mutant Mice Are Not Enhanced by L1 Function-Triggering Reagents

To investigate the function of the L1-70 fragment, which was implicated in L1-mediated neurite outgrowth, neuronal survival and neuronal migration [28], we created gene-edited mice and showed that in these L1/858-863 and L1/687 mice the L1-70 fragment was absent or was only present in marginal amounts [30] (see also the scheme in Supplementary Figure S1). In addition, we generated L1-201 mice with normal L1 processing but an L1 syndrome-like phenotype (see also the scheme in Supplementary Figure S1). Here, we aimed to analyze the consequence of the lack of L1-70 on neuronal and glial function in the L1/858-863 and L1/687 mutants, and on mitochondrial characteristics in all three mutant lines.

**Figure 1:**
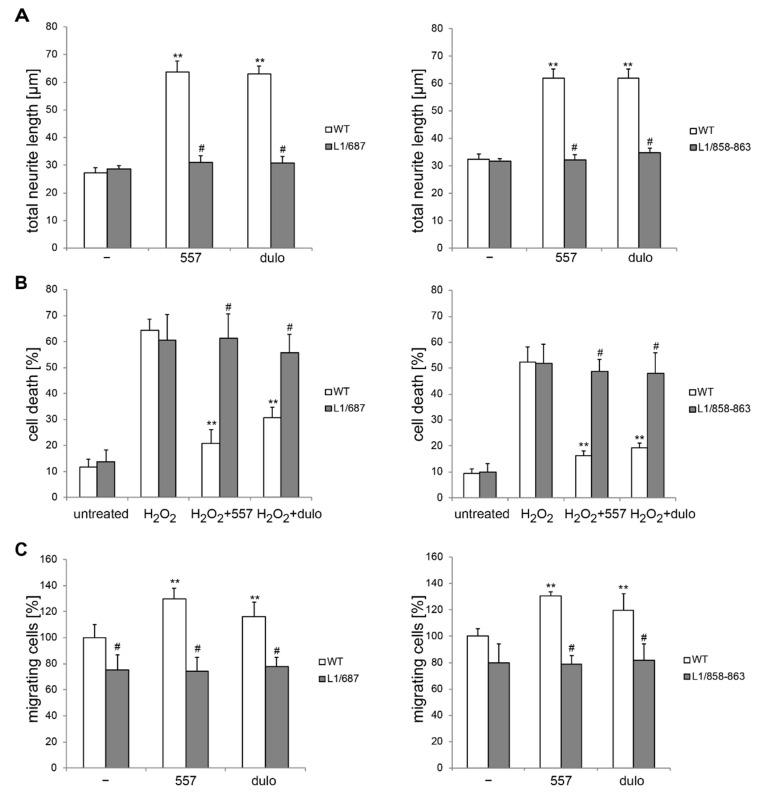
Cerebellar neurons from L1/858–868 and L1/687 mice do not respond to treatment with antibody 557 or duloxetine. Cerebellar neurons and explants were prepared from 6- to 7-day-old male wild-type (WT, white bars) and L1 mutant littermate (gray bars) mice and maintained on PLL substrate. (**A**) Neurons were left untreated (−) and treated with 50 µg/mL of antibody 557 (557) or 100 nM of duloxetine (dulo) for 24 h, and neurite outgrowth was determined from 100 cells per genotype and condition for each experiment. (**B**) Cell death was measured in the absence (untreated) or presence of 10 µM hydrogen peroxide (H_2_O_2_) and 50 µg/mL of antibody 557 (H_2_O_2_+557) or 100 nM of duloxetine (H_2_O_2_+dulo) for 24 h. Cells were stained with calcein and propidium iodide, and cell death was measured by counting live and dead cells from three wells per genotype and treatment. (**C**) After 16 h of culturing, explants were left untreated (−) or treated for 24 h with 50 µg/mL of antibody 557 (557) or 100 nM of duloxetine (dulo). Migrating cells from 12 explants per condition and genotype were counted. (**A**–**C**) Values show the means ± SD from three (**B**,**C**) or four (**A**) independent experiments. ** *p* < 0.01 difference relative to PLL, # *p* < 0.05 difference relative to the wild-type (two-way ANOVA with Tukey’s post-hoc test).

First, we investigated neurite outgrowth as well as neuronal survival and migration in cultured cerebellar granule neurons from L1/858-863 and L1/687 mutants maintained on poly-L-lysine (PLL). On this substrate, the neurite outgrowth and survival of neurons from mutant mice were comparable with those from wild-type neurons (Figure 1A,B). Next, we cultured neurons on PLL and treated them with the function-triggering L1 antibody 557, which binds to a sequence stretch in the third fibronectin type III (FNIII) domain (see the scheme in Supplementary Figure S1), or the L1 mimetics duloxetine, piceid, honokiol and crotamiton. Treatments with antibody 557 and L1 mimetics enhanced L1 expression, generated L1-70 and stimulated L1-triggered signaling [32,33]. In contrast to wild-type neurons, which had higher total neurite lengths and enhanced survival after antibody 557 and L1 agonist treatment compared with control levels, neither neurite outgrowth nor neuronal survival of L1/858-863 and L1/687 cells was stimulated by antibody 557 or the L1 agonists duloxetine (Figure 1A,B), piceid, honokiol or crotamiton (Supplementary Figure S2A,B). The migration of mutant neurons on PLL was reduced compared with that of the wild-type littermate neurons and could not be enhanced by treatment with antibody 557 or duloxetine, which enhanced the migration of the wild-type littermate neurons (Figure 1C). These results suggest that L1-70 is not involved in neuronal survival or neuritogenesis under basal conditions, but is required for L1 agonist-mediated neuritogenesis, and neuronal survival and migration. In contrast, neuronal migration under non-stimulated conditions seems to depend, to some degree, on the presence of L1-70.

**Figure 2:**
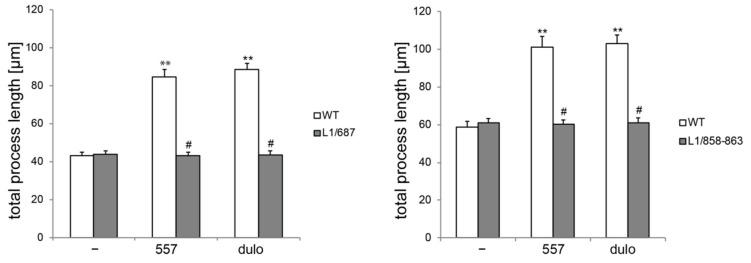
Schwann cells from L1/858–868 and L1/687 mice were not stimulated by antibody 557 or duloxetine. Schwann cells were prepared from 6- to 7-day-old male wild-type (WT, white bars) and L1 mutant littermate (gray bars) mice and seeded on a PLL substrate. Process lengths were determined after treatment without (−) or with 50 µg/mL of antibody 557 (557) or 100 nM of duloxetine (dulo) for 24 h. Values were obtained from three independent experiments analyzing 100 cells per genotype and condition for each experiment. Values show the means ± SD. ** *p* < 0.01 difference relative to PLL, # *p* < 0.05 difference relative to the wild-type (two-way ANOVA with Tukey´s post-hoc test).

We next determined the consequences of the absence of L1-70 for Schwann cell function, since Schwann cells also express L1, and L1 was shown to be involved in Schwann cell axon interactions [34,35]. The process length of Schwann cells without L1-70 was normal under non-stimulated conditions but was not enhanced by treatment with antibody 557 or L1 agonists, which enhance the length of the processes of wild-type Schwann cells (Figure 2 and Supplementary Figure S3).

These results distinguish the neurons and Schwann cells from the L1-70-lacking mice from the cells of the L1-201 mutant, which could be stimulated by function-triggering L1 antibody 557 and L1 mimetics, but were impaired under basal conditions [31].

### 2.2. Impaired Mitochondrial Function in Neurons from L1/858-863, L1/687 and L1-201 Mice

**Figure 3:**
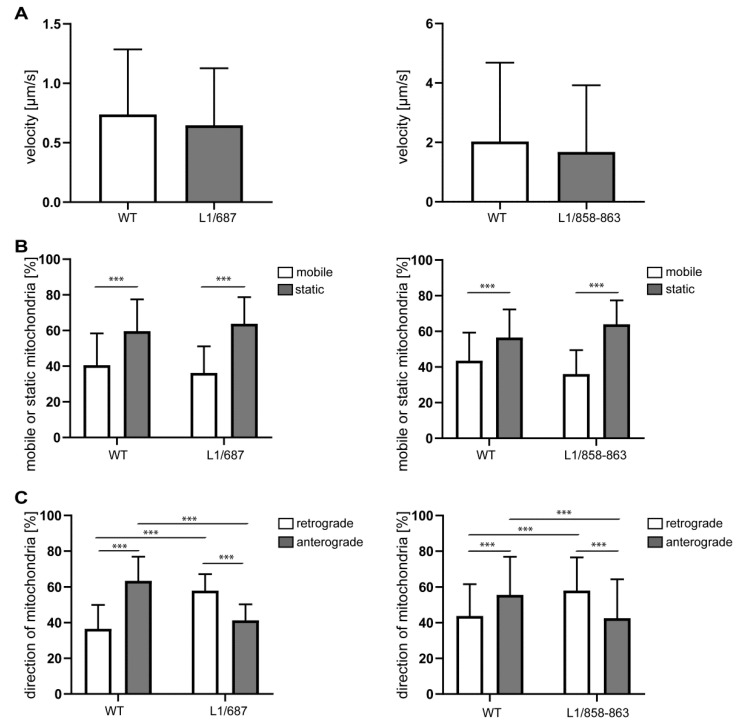
Normal velocity and mobility but enhanced retrograde transport in mitochondria from L1/687 and L1/858-863 mice. Mitochondrial velocity (**A**), mobility (**B**) and transport direction (**C**) were analyzed in cultured cerebellar neurons from 7-day-old male mutants and wild-type littermate mice. Means ± SD from three independent experiments are shown. *** *p* < 0.005 (velocity: Mann–Whitney test; mobility and direction of L1/868-863: Kruskal–Wallis test with Dunn’s multiple comparison test; mobility and direction of L1/687: two-way ANOVA with Tukey´s post-hoc test).

**Figure 4:**
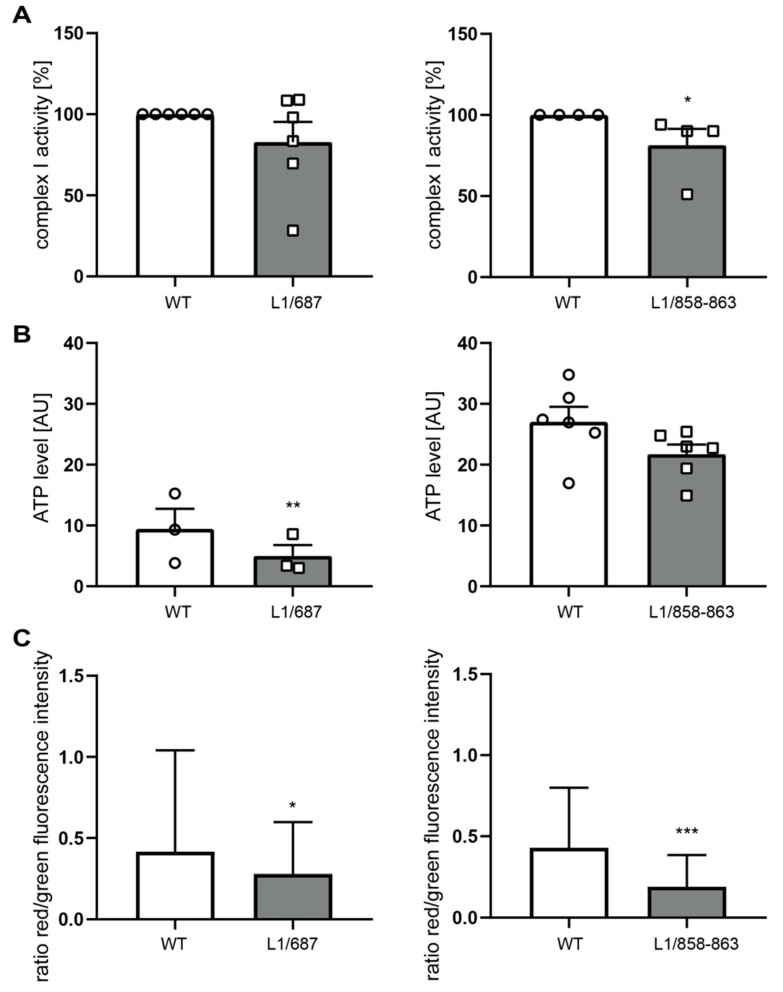
Mitochondria from L1/687 and L1/858-863 mice show a reduced membrane potential. (**A**) Mitochondrial Complex I activity was determined using isolated mitochondria from whole brains of 7- to 9-day-old male L1/687, L1/858-863 (gray bars) and wild-type (WT, white bars) littermate mice. (**B**,**C**) Mitochondrial ATP levels (**B**) and mitochondrial membrane potential (**C**) were determined in cultured cerebellar neurons from 7-day-old male L1/687, L1/858-863 (gray bars) and wild-type littermate (WT, white bars) mice. Means ± SD (**C**) or means ± SEM and values from individual mice (**A**,**B**) are shown; L1/687: n = 6 for Complex I activity, n = 3 for ATP level and membrane potential; L1/858-863: n = 4 for Complex I activity, n = 6 for ATP levels and n = 3 for membrane potential. * *p* < 0.05, ** *p* < 0.01, *** *p* < 0.005 (Mann–Whitney test).

We next analyzed mitochondrial activity and transport in neurons from L1/858-863, L1/687 and L1-201 mutant mice. In L1-70-lacking L1/858-863 and L1/687 neurons, the velocity and mobility of the mitochondria was normal, but the mitochondria moved more retrogradely than the mitochondria from wild-type neurons (Figure 3A–C). The activity of Complex I, which is the largest multi-subunit complex of the respiratory chain, showed a slight tendency to be reduced in neurons from L1/687 mice, and mitochondrial ATP levels were lower than in wild-type neurons. However, in neurons from L1/858-863 mice, Complex I activity was reduced, and ATP levels tended to be reduced. Furthermore, L1/687 and L1/858-863 mutant neurons exhibited a lower mitochondrial membrane potential compared with those from the wild-type littermates (Figure 4A–C).

**Figure 5:**
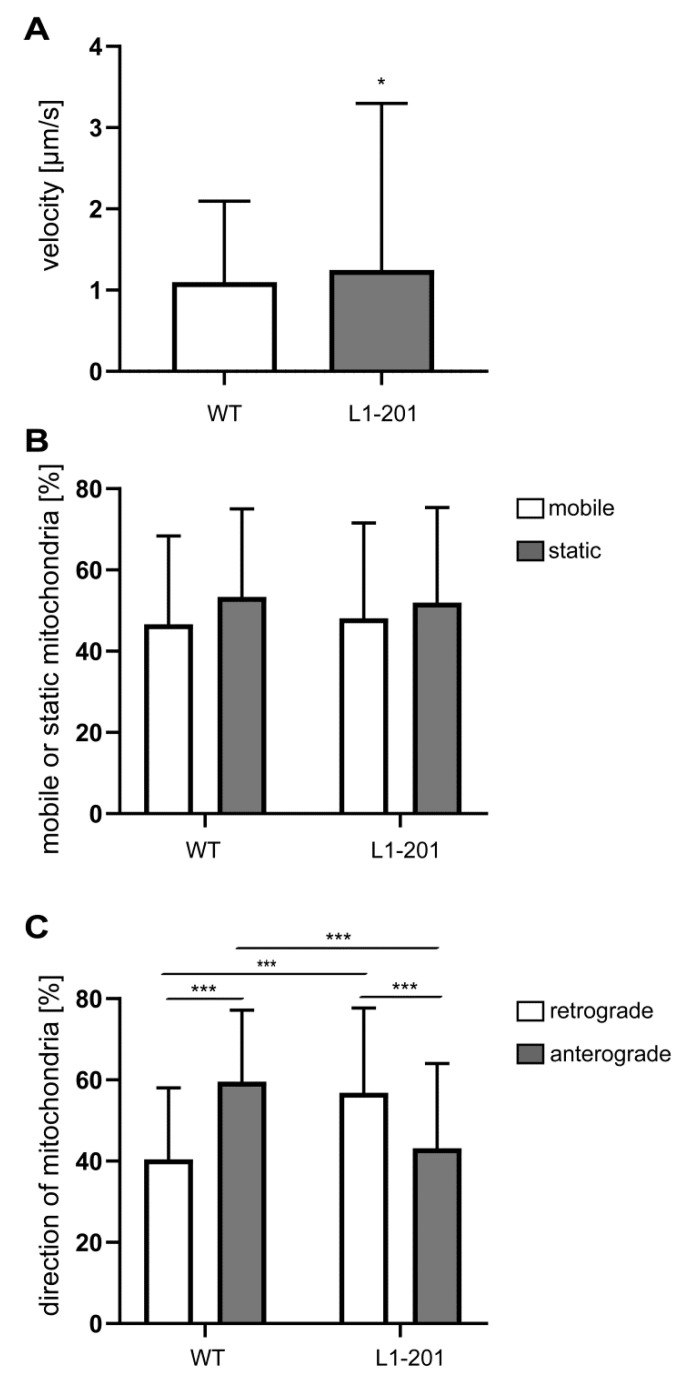
Mitochondria from L1/201 mice showed normal mobility and enhanced velocity but moved more retrogradely than mitochondria from wild-type neurons. Mitochondrial velocity (**A**), mobility (**B**) and transport direction (**C**) were analyzed in cultured cerebellar neurons from 7-day-old male L1-201 mice and wild-type littermates. Mean values ± SD are shown; n = 3. * *p* < 0.05, *** *p* < 0.005 (velocity: Mann–Whitney test; mobility: two-way ANOVA with Tukey´s post-hoc test; direction: Kruskal–Wallis test with Dunn’s multiple comparison test).

Mitochondrial functions of L1-201 mutant mice—in which the L1-70 fragment is generated—showed impaired neuronal migration, enhanced cell death and a reduced process length of Schwann cells on the PLL substrate but enhanced process length, and enhanced migration and survival after L1 agonist treatment [31]. Interestingly, neurons from L1-201 mutant mice showed differences in mitochondrial functions compared with wild-type neurons and to the neurons from mutant mice lacking L1-70. The velocity of L1-201 mitochondria was higher than that of wild-type and L1-70-lacking neurons, while the mobility of L1-201 mitochondria was comparable with that of wild-type mitochondria (Figure 5A,B). Furthermore, L1-201 mitochondria, similar to L1-70-lacking mitochondria, also moved more retrogradely than wild-type mitochondria (Figure 5C).

**Figure 6:**
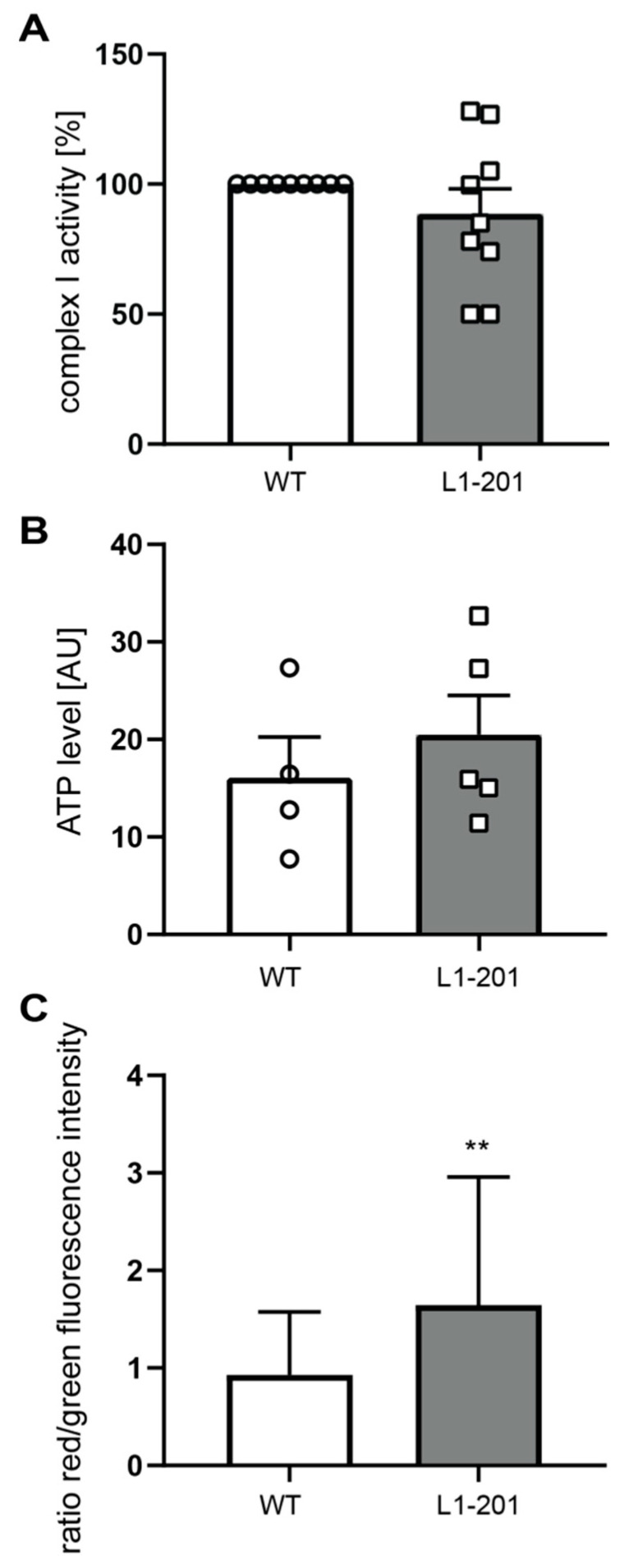
Normal Complex I activity, normal ATP levels and higher membrane potential in mitochondria from L1-201 mice. (**A**) Mitochondrial Complex I activity was determined using isolated mitochondria from whole brains of 7- to 9-day-old male L1-201 (gray bars) and wild-type (WT, white bars) littermate mice (n = 9 mice per group). (**B**,**C**) Mitochondrial ATP levels (**B**) and mitochondrial membrane potential (**C**) were determined in cultured cerebellar neurons from 7-day-old male L1-201 (gray bars) and wild-type (WT, white bars) littermate mice (n = 4 mice per group). Means ± SEM and values from individual mice are shown for Complex I activity and ATP levels (**A**,**B**) and means ± SD for membrane potential (**C**); n = 3. ** *p* < 0.01 (membrane potential: Student´s *t*-test; Complex I activity and ATP levels: Mann–Whitney test).

**Figure 7:**
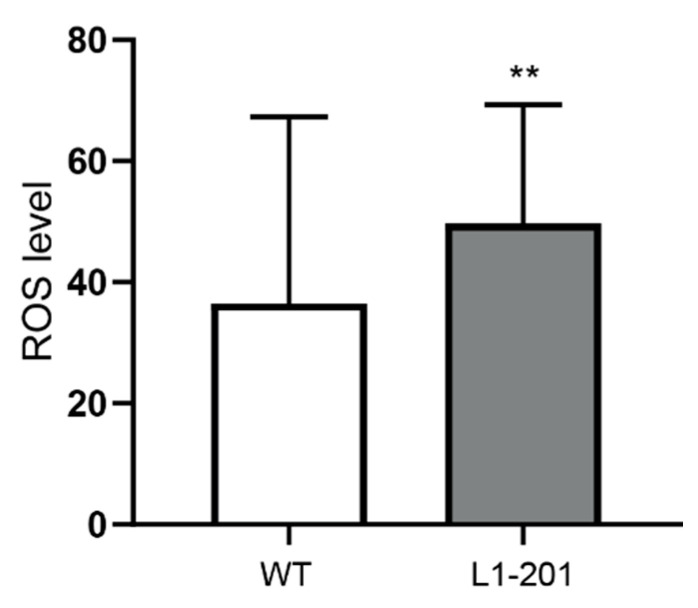
Enhanced levels of ROS in neurons from L1-201 mice. ROS levels were determined in cultured cerebellar granule cells from 6- to 7-day-old male L1-201 mice and their wild-type male littermates. Means ± SD are shown; n = 3. ** *p* < 0.01(Mann–Whitney test).

Complex I activity and ATP levels were normal in L1-201 mitochondria, whereas the membrane potential was higher in L1-201 mitochondria compared with that of wild-type neurons (Figure 6A–C). Furthermore, ROS levels in neurons from L1-201 mutant mice were enhanced (Figure 7), showing that these neurons are differently impaired in comparison with neurons lacking L1-70.

Our results show that L1/687 and L1/858-863 mice exhibit lower Complex I activity, reduced mitochondrial membrane potential and reduced ATP levels, whereas their mitochondria move more retrogradely and their velocity and mobility are normal. This indicates that the generation of the L1-70 fragment is important for mitochondrial Complex I activity, production of ATP and generation of normal mitochondrial membrane potential, but not for mitochondrial velocity or mobility. A comparison with mitochondria from L1-201 mice showed that these mitochondria also move more retrogradely, suggesting that the absence of L1-70 is not responsible for the retrograde movement of mitochondria from L1/687 and L1/858-863 mutant neurons, but is indicative of stressed or unhealthy mitochondria in these mutants.

## 3. Discussion

Fragments of L1 were previously shown to be imported into the cytosol and from there into intracellular organelles such as the mitochondria and the nucleus [28,36,37]. In mitochondria, L1-70 interacts with the Complex I subunit NADH dehydrogenase ubiquinone flavoprotein 2. L1-deficient neurons showed reduced Complex I activity and impaired mitochondrial membrane potential, suggesting that L1 and especially the L1-70 fragment regulate mitochondrial functions [28]. To substantiate this notion and to determine the function of the L1-70 fragment, we analyzed mice which expressed full-length L1 but not the L1-70 fragment. Thus, it was possible to specifically address the question about which mitochondrial processes the L1-70 fragment functions in. Our results showed that the velocity and mobility of mitochondria were not affected by L1-70 but that the absence of L1-70 led to reduced Complex I activity, membrane potential and ATP production. In contrast, Complex I activity and ATP production were normal and mitochondrial membrane potential was enhanced in mice expressing L1 with the D201N mutation, demonstrating that the absence of L1-70 is indeed responsible for reduced Complex I activity, membrane potential and ATP production, and highlighting that not all L1 mutations lead to a similar phenotype. Insufficient energy supply at the synapses and growth cones could cause the defects seen during nervous system development and in the brain function of adult L1-deficient mice and mice in which the L1-70 fragment is absent.

The mitochondria of neurons in which L1-70 is absent and the mitochondria of neurons with the D201N mutation moved more retrogradely than anterogradely, which indicates that the absence of L1-70 is not responsible for the enhanced retrograde transport but other functions of L1 are responsible, such as L1 signal transduction or the interaction of full-length L1 with its binding partners.

Previous studies showed that treatment of wild-type cerebellar neurons with the function-triggering antibody 557 and the L1 mimetics duloxetine, piceid, tacrine, ethinyl estradiol, crotamiton and honokiol enhanced the expression of L1, stimulated the generation of L1-70 and enhanced neuritogenesis as well as neuronal migration and survival [32,33]. Schwann cell proliferation, migration and myelination were also enhanced by antibody 557 and L1 mimetics. In addition, the phosphorylation of Erk1/2 was higher in neurons treated with antibody 557 and L1 mimetics than in control neurons [32]. These results suggest that L1 expression, L1 proteolysis and L1 signaling are enhanced by treatments with antibody 557 and L1 mimetics.

Neurons from mice without L1-70 exhibited reduced neurite outgrowth and neuronal survival and migration when the cells were stimulated with antibody 557 or L1 agonists, but were not affected without stimulation. This feature also distinguishes L1-70-deficient neurons from L1-201 neurons with impaired basal neuronal functions but susceptibility to stimulation by antibody 557 or L1 agonists [31]. These results confirm previous findings that the generation of L1-70 from full-length L1 is important for L1-mediated neurite outgrowth and neuronal migration. However, we have shown that L1-70 is not essential for these features when L1 is not triggered.

After showing that L1 plays important roles in the functions of mitochondria [28], we investigated the L1–mitochondria relationship in more detail. Mitochondria are essential for neural energy metabolism, since they produce the fuel needed for processes such as neuronal proliferation and migration, neurite outgrowth, neurotransmission, synapse formation and synaptic plasticity. Regions with high energetic requirements, such as growth cones and presynaptic terminals, contain more mitochondria than other cellular compartments. Mitochondria are transported anterogradely from the cell soma to distal axons and synaptic terminals, where they remain stationary to support synaptic transmission by supplying ATP and buffering Ca^2+^. When mitochondria are aged or damaged, they are retrogradely transported back to the cell soma to be degraded [19]. Throughout their movements in the axon, mitochondria can quickly switch between anterograde and retrograde movement, and their net direction in cultured neurons results primarily from the modulation of the fraction of time spent moving anterogradely [38]. Neurons from the three L1 mutants tested in the present study included more retrogradely moving mitochondria, suggesting that these mitochondria were less healthy. In addition, mitochondrial membrane potential and ATP levels were reduced in neurons without L1-70, features which might cause reduced neuronal migration and neurite outgrowth when cells are stimulated. This view is supported by the fact that mitochondrial membrane potential and ATP production in L1-201 neurons with L1-70 were normal and that neurite outgrowth and neuronal migration could be stimulated. Of note, besides being a major metabolic center for catabolism, the mitochondria are also an important source of the metabolites required for the biosynthesis of lipids, carbohydrates, proteins, amino acids and nucleotides [5]. Deficiencies in these molecules in the L1-70-deprived mutants might also contribute to the impairments in neuronal functions seen in these mutants and in L1-deficient mice.

When their function is impaired, the mitochondria contribute to severe pathologies of the nervous system, not only due to metabolic insufficiency but also to the production of ROS and activation of caspases [1,11,39], with the highest basal rate of ROS production being in the brainstem and cerebellum [40]. Here, we observed higher ROS levels and enhanced cell death of cerebellar neurons from L1-201 mice, which suggests that the oxidative stress and subsequent death of these neurons is related to their impaired mitochondrial function. Interestingly, so far, we have not observed higher numbers of dead cells in the brain in the absence of L1-70 or in L1-deficient brains. We expect, however, that our novel in vitro results might warrant a closer investigation of stressed cells in the cortex and cerebellum of these mutant mice. Further interesting observations in the context of mitochondrial activities are that the mitochondria move differently in radially versus tangentially migrating interneurons in the cerebellum [41]. It remains to be seen which adhesion molecules operate in these two different types of migration.

## 4. Materials and Methods

### 4.1. Mice

Gene-edited mice expressing L1 with an arginine-to-alanine exchange at Position 867 in the first FNIII domain (L1/687 mutant mice), with a mutation of the dibasic sequence RKHSKR to SKHSSS at Positions 858–863 in the third FNIII domain (L1/858-863 mutant mice) [30] and with an aspartic acid to asparagine exchange at Position 201 (L1-201 mutant mice) [31] have been described. Mice were bred and maintained at the Universitätsklinikum Hamburg-Eppendorf at 21 °C on a 12 h light/12 h dark cycle with ad libitum access to food and water. L1-201, L1/687 or L1/858-863 males and their respective male wild-type littermates were used for all experiments. Experiments were approved by the local authorities of the State of Hamburg (Behörde für Justiz und Verbraucherschutz der Freien und Hansestadt Hamburg, Amt für Verbraucherschutz, Lebensmittelsicherheit und Veterinärwesen; animal permit numbers ORG 1022 (approval date 31 July 2020), N19/004_ZuchtNeuro (approval date 7 March 2019)). The experiments were designed, and the manuscript was prepared according the ARRIVE guidelines [42].

### 4.2. Cerebellar Neuron Culture, Neurite Outgrowth, Neuronal Migration and Neuronal Survival

Cultures of dissociated cerebellar granule neurons were prepared from 6- to 7-day-old male mice as described [43]. In each experiment, two or three mice per genotype were used. For determining the neurite outgrowth, cells were seeded at a density of 3.75 × 10^4^ cells/well in 0.01% poly-L-lysine (PLL; Sigma-Aldrich, Taufkirchen, Germany)-coated 48-well tissue culture plates (ThermoFisher Scientific, Waltham, MA, USA) in a defined serum-free medium (Neurobasal A (ThermoFisher Scientific) supplemented with 2 mM L-glutamine, 4 nM L-thyroxine, 1 mg/mL BSA, 12.5 μg/mL insulin, 30 nM sodium selenite, 100 μg/mL transferrin, 0.1 mg/mL streptomycin and 10 U/mL penicillin (all Sigma-Aldrich)) and allowed to settle for 30 min. They were then treated with 50 µg/mL of L1 function-triggering antibody 557 directed against amino acids 817–835 at the transition between the second and third FNIII-like domains ([33]; no RRID available) or 100 nM of the L1 agonists duloxetine (Tocris Bioscience, Bristol, UK; catalog #4798/10), crotamiton (Santa Cruz Biotechnology, Heidelberg, Germany; catalog #sc-205639), honokiol (Tocris Bioscience; catalog #4590/10) and piceid (Santa Cruz Biotechnology; catalog #sc-203203) [32] for 24 h. After fixing and staining with 1% toluidine blue and 1% methylene blue in 1% sodium tetraborate, images were taken and total neurite lengths from at least 100 cells per experiment were quantified using an AxioObserver.A1 microscope (Carl Zeiss, Oberkochen, Germany) with a 20× objective (aperture 0.4) and AxioVision 4.6 software (Carl Zeiss) [43]. To determine cell death, cerebellar granule neurons were seeded at a density of 1 × 10^6^ cells/mL in PLL-coated 48-well tissue culture plates (ThermoFisher Scientific) and maintained overnight in a defined serum-free medium as indicated above [43]. Cells were then treated with 50 µg/mL of antibody 557 or 100 nM of duloxetine, then immediately exposed to oxidative stress by the addition of 10 µM H_2_O_2_ for 24 h. Live and dead cells were then stained with calcein-AM (ThermoFisher Scientific) and propidium iodide (Sigma-Aldrich) and imaged with a Zeiss AxioObserver.A1 microscope (Carl Zeiss) with a 20× objective (aperture 0.4) and AxioVision 4.6 software (Carl Zeiss) [43]. Live and dead cells were counted in four images (containing 350–400 cells each) from each of three wells per condition and experiment using ImageJ (version 1.53; https://imagej.nih.gov/ij/index.html; RRID:SCR_003070, 8 March 2022). For determining cell migration, cerebellar explants were prepared from two to three 6- to 7-day-old male mice per genotype and experiment as described, and the migration of neurons was determined from 12–15 explants per condition and experiment [44]. Briefly, cerebella were consecutively forced through nylon nets with pore sizes of 300, 200 and 100 μm (VWR, Darmstadt, Germany). Tissue pieces were plated on glass coverslips coated with PLL and maintained for 16 h in a culture medium as indicated above containing 20% horse serum (ThermoFisher Scientific). The culture medium was then replaced by serum-free medium, and 50 µg/mL of antibody 557 or 100 nM of duloxetine was added for 32 h. Explants were then fixed and stained with 1% toluidine blue and 1% methylene blue in 1% sodium tetraborate. To quantify the number of migrated cells, explants were imaged with a Zeiss AxioObserver.A1 microscope (Carl Zeiss) with a 5× objective (aperture 0.1), and all cells outside of the explant border were counted using ImageJ software [45]. Experiments were carried out in duplicate or triplicate and performed independently three times.

### 4.3. Schwann Cell Process Length

Schwann cells were isolated from male 6- to 7-day-old mice as described [46]. In each experiment, two or three mice per genotype were used. For determination of the Schwann cell process length, cells were seeded in a serum-free medium (DMEM/Ham´s F12 1:1, 2 mM L-glutamine, 60 ng/mL progesterone, 16 mg/mL putrescine, 5 mg/mL insulin, 0.4 mg/mL L-thyroxine, 160 ng/mL sodium selenite, 10 ng/mL triiodothyronine, 38 ng/mL dexamethasone, 100 U/mL penicillin and 100 mg/mL streptomycin; all from Sigma-Aldrich) at a density of 1 × 10^4^ cells/well into PLL-coated 48-well tissue culture plates (ThermoFisher Scientific) and allowed to settle for 30 min. Cells were then treated with 50 µg/mL of antibody 557 or 100 nM of duloxetine for 24 h. After fixing and staining with 1% toluidine blue and 1% methylene blue in 1% sodium tetraborate, images of the process were taken, and the lengths of the process from at least 100 cells per condition and experiment were quantified using an AxioObserver.A1 microscope with a 20× objective (aperture 0.4) and AxioVision 4.6 software (Carl Zeiss) [43]. Experiments were carried out in duplicate and performed independently three times.

### 4.4. Mitochondrial Movement

Mitochondria were labeled with MitoTracker^®^ Red CMXRos (ThermoFisher Scientific; catalog #M7512). A MitoTracker stock solution (1 mM) was diluted to a final concentration of 50 nM in the pre-warmed cerebellar neuron culture medium. Cerebellar granule neurons (density: 1−2 × 10^6^ cells/well of a 6-well plate containing a 20 mm glass coverslip) were prepared as stated above and cultured for two to three days in a medium as indicated above containing 5% horse serum. The medium was then replaced with a serum-free medium, and the cells were incubated with 50 nM of MitoTracker for 30 min at 37 °C. This solution was then replaced with fresh serum-free medium, and the cells were analyzed at 37 °C under 5% CO_2_ by time-lapse imaging using an upright Nikon Eclipse Ti microscope (Nikon Instruments, Amsterdam, The Netherlands) combined with spinning disk (Visitron, Puchheim, Germany) live-cell confocal technology (Visitron Systems) and a 100× objective (aperture 1.45). For live cell imaging, a 561 nm argon laser was used at 10% intensity, and images were taken at intervals of 2 s for a duration of 5 min. Videos were acquired using the VisiView 5.0 software (Visitron Systems). To determine mitochondrial velocity, mobility and the direction of mitochondrial transport (anterograde or retrograde), we used the kymograph (time space plot) plugin and the velocity tool from ImageJ (available online: https://www.embl.de/eamnet/html/body_kymograph.html, 8 March 2022). The kymograph represents a time and space plot: the *x*-axis indicates the distance, and the *y*-axis shows the time. Vertical lines represent no movement, while diagonal lines show mobile mitochondria. With respect to the position of the cell soma, the direction (right or left) of the diagonal lines indicates retrograde or anterograde transport, respectively [47]. Mitochondrial motility, direction and mobility were analyzed as described [28,47]. For each experiment, one mouse per genotype was used. Experiments were carried out in duplicate and performed independently three times; 50 (L1/687) or 75 (L1-201 and L1/858-863) mitochondria were evaluated for velocity and direction, and 30 (L1/687) or 45 (L1-201 and L1/858-863) kymographs were evaluated for mobility.

### 4.5. Mitochondrial Membrane Potential

The dual-emission potential-sensitive probe JC-1 iodide (1,1′,3,3′-tetraethyl-5,5′,6,6′-tetrachloroimidacarbocyanine iodide; Santa Cruz Biotechnology; catalog #sc-364116) was used to measure the membrane potential of the mitochondria [48]. JC-1 enters the mitochondria and shows potential-dependent accumulation: at higher mitochondrial membrane potentials, JC-1 forms red-fluorescent “J-aggregates”, while in cells with a lower mitochondrial membrane potential, it leaks out of mitochondria and is present as fluorescent green monomers in the cytosol. The ratio of red to green fluorescence of JC-1 is dependent only on the mitochondrial membrane’s potential and is not influenced by mitochondrial size, shape or density in the cell.

To determine the mitochondrial membrane potential, cerebellar granule cells were prepared as stated above and plated at a density of 1−2 × 10^6^ cells/mL onto 22 µm glass coverslips coated with PLL and cultured in a serum-free medium for two days. Afterwards, cells were stained with 3 µM of JC-1 in Hank´s balanced salt solution (HBSS; Sigma-Aldrich) for 30 min, incubated with HBSS alone for 15 min and then observed under a spinning disk microscope with a 60× objective (aperture 1.4). For detecting mitochondria with high mitochondrial membrane potential, a rhodamine filter set was used together with the fluorescein filter for detecting depolarized mitochondria with low mitochondrial membrane potential. Experiments were carried out in duplicate and performed independently three times using one mouse per genotype and experiment. The total cell fluorescence intensity was calculated for the green and red channels separately, and the red to green fluorescence intensity ratio was determined from 10 neurons per image and 10 images per group and experiment.

### 4.6. Mitochondrial Complex I Activity

Whole brains were isolated from 6- to 8-day-old male mice, frozen in liquid nitrogen and stored at −80 °C until use. For isolation of the mitochondria, the Mitochondria Isolation Kit for Tissue from ThermoFisher Scientific (catalog #89801) was used. For determining Complex I activity, mitochondria were applied to the MitoCheck^®^ Mitochondrial Complex I Activity Assay Kit (Cayman Chemical, Ann Arbor, MA, USA; catalog #700930). Experiments were carried out in duplicate and performed independently three times using four (L1/858-863), six (L1/687) or nine (L1-201) mice in total per genotype. Only Complex I activity was determined in the present study, since we had found that other complexes’ activities were not altered by the L1-70 fragment [28].

### 4.7. ATP Levels

ATP levels were determined using the BacTiter-Glo^®^ 2.0 Cell Viability Assay (Promega, Walldorf, Germany; catalog #G8230) according to the manufacturer´s protocol. In brief, cerebellar granule cells were cultured in a serum-free medium as indicated above at a density of 2.5 × 10^5^ cells/well in 48-well plates for 2 days. Afterwards, cells were scraped off into 200 µL of serum-free medium, 100 µL of the supernatant was transferred into white 96-well plates and 100 µL of BacTiter-Glo was added to the wells. Plates were incubated for 5 min at room temperature with gentle shaking and then the luminescence was measured using a luminometer (Mithras LB943; Berthold Technologies, Bad Wildbad, Germany). ATP concentrations were determined using an ATP standard curve. Experiments were carried out three times independently in triplicate using three (L1/687), five (L1-201) and six (L1/858-863) mice in total per genotype.

### 4.8. Reactive Oxygen Species

Intracellular ROS were monitored using the ROS-sensitive fluorescent probe 2′,7′-dichlorofluorescin diacetate (DCFH-DA; Biomol, Hamburg, Germany; catalog #Cay85155-50), which penetrates into cells rapidly and is hydrolyzed by ROS into a fluorescent green dye. Cerebellar granule cells from 6- to 7-day-old mice (one mouse per genotype and experiment) were prepared as stated above and seeded onto glass coverslips as mentioned above, maintained in the culture for 3 days in a serum-free medium and stained with 10 µM of DCFH-DA for 30 min at 37 °C and 5% CO_2_. The medium was then replaced by HBSS without phenol red, and cells were imaged under a spinning disk microscope with a 40× objective (aperture 1.2) and with an excitation wavelength of 488 nm. Experiments were carried out in duplicate and performed independently three times; for each condition and experiment, 10 images were taken for analysis.

### 4.9. Statistics

All numerical data are presented as group mean values with the standard error of the mean (SEM) or standard deviation (SD); for small sample numbers, individual data are additionally indicated. The normal distribution of the data was determined using the Shapiro–Wilk test or the D’Agostino–Pearson test. For data with a normal distribution, Student’s *t*-test was used to compare two groups, whereas for comparisons of more than two groups, we performed ANOVA followed by Tukey’s post-hoc test for multiple comparisons. Groups containing small sample numbers or data that were not normally distributed were analyzed using the Mann–Whitney U-test for comparing two groups or the Kruskal–Wallis test by ranks followed by Dunn’s multiple comparison test when there were more than two groups. The statistical tests used for comparisons are indicated in the figure legends. Analyses were performed using SPSS (version: 26; RRID:SCR_002865), SigmaPlot (version: 14; RRID:SCR_003210) or GraphPad (version: 8; RRID:SCR_000306) software.

## Data Availability

Data and materials will be made available upon reasonable request.

## Supplementary Materials

The following are available online at https://www.mdpi.com/article/10.3390/ijms23084337/s1.
