# Supplementary material for: Mitochondrial and Neuronal Dysfunctions in L1 Mutant Mice
Source: Int J Mol Sci. 2022 Apr 14;23(8):4337. doi: 10.3390/ijms23084337 (PMC9026747; doi:10.3390/ijms23084337)
Supplement: Supplementary file 1 [file ijms-23-04337-s001.zip › ijms-1653668-supplementary.pdf]

Supplementary information

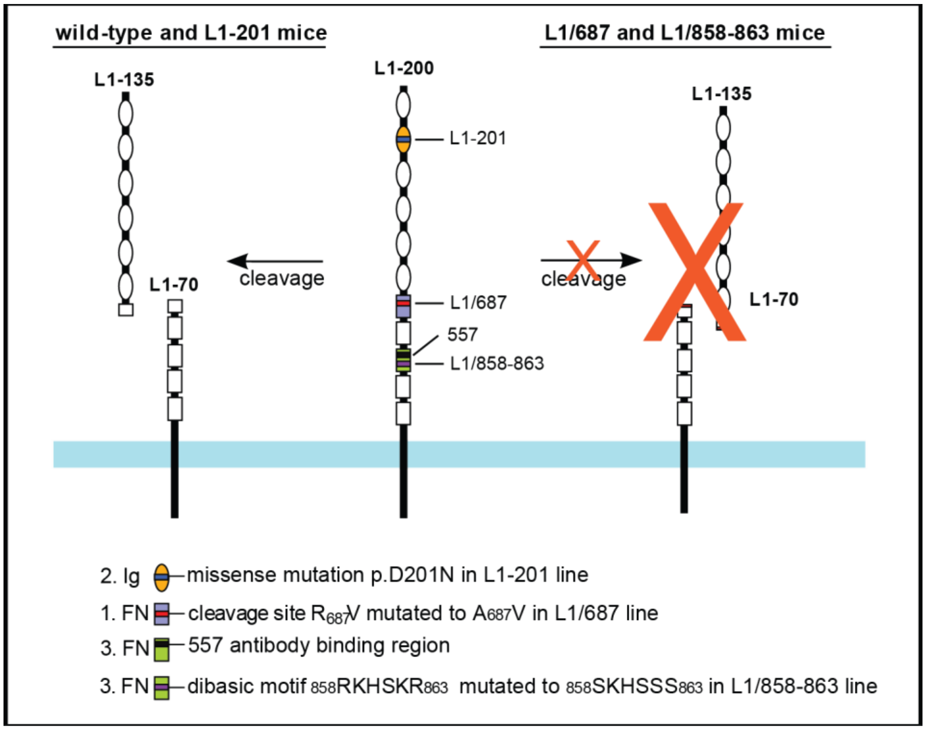

Supplementary Figure S1: Schematic presentation of L1 in the mouse lines used. L1 consists of six inmunoglobulin (Ig)-like (ovals) and five fibronectin type III (FNIII) (rectangles) extracellular domains, a transmembrane domain passing the plasma membrane (blue) and an intracellular tail. In the L1-201 line the missense mutation D201N was introduced into the second Ig-like domain [31]. The sequence $R_{687}V$ in the first FNIII domain was changed to $A_{687}V$ in the L1/687 mouse line and $_{858}RKHSKR_{863}$ in the third FNIII domain was changed to $_{858}SKHSSS_{863}$ in the L1/858-863 mouse line [30]. Cleavage of full-length L1 (L1-200) occurs in wild-type and L1-201 mice generating a soluble 135 kDa fragment (L1-135) and a membrane-bound 70 kDa fragment (L1-70) (left side), whereas in the L1/687 and L1/858-863 lines these fragments are not generated (right side). The epitope for the L1 function-triggering antibody 557 is indicated in the third FNIII domain.

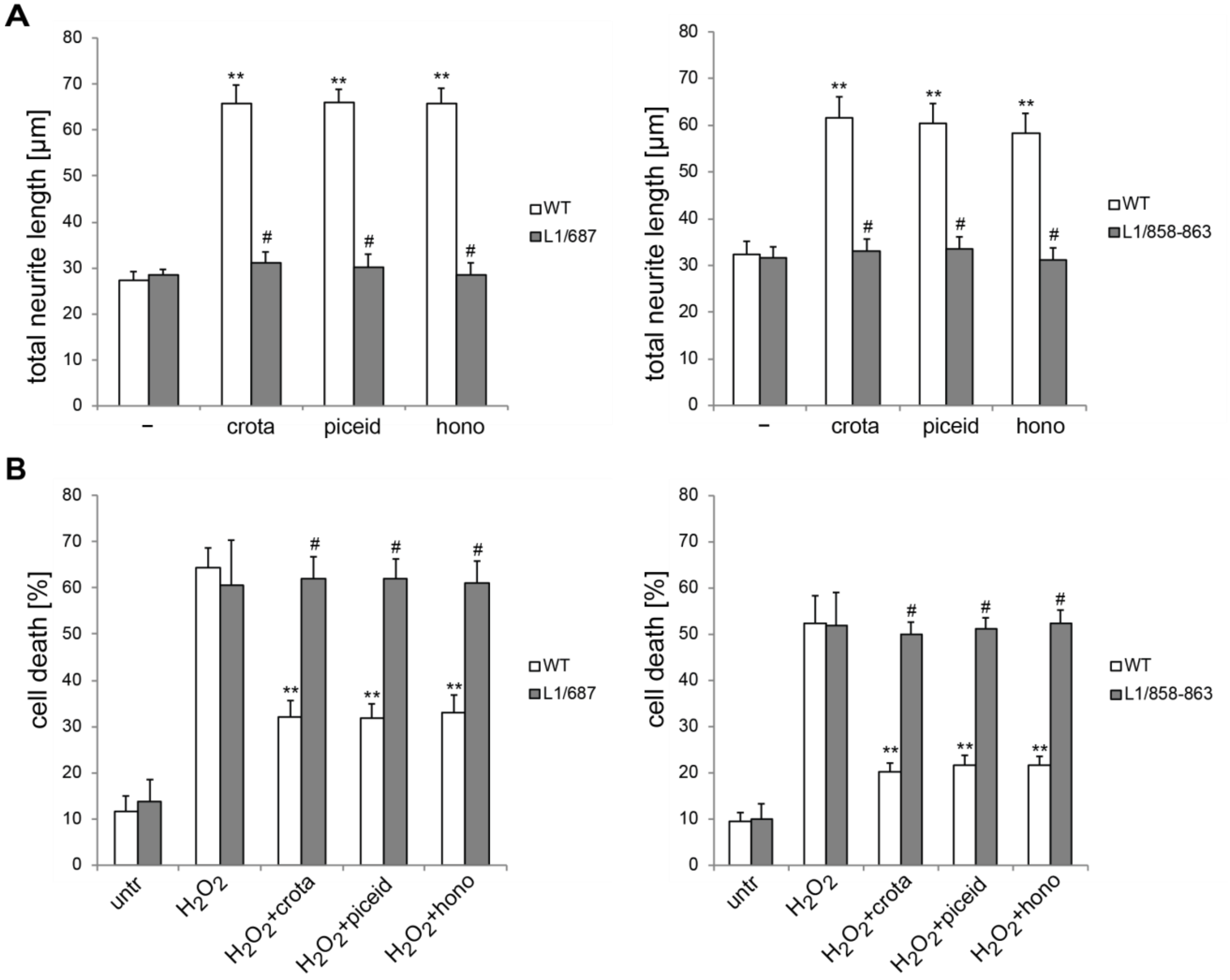

Supplementary Figure S2. Cerebellar neurons from L1/687 and L1/858-868 mice do not respond to stimulation with L1 agonists. Cerebellar neurons and explants were prepared from 6- to 7-day-old male wild-type (WT, white bars) and male L1 mutant littermate (gray bars) mice and maintained on a PLL substrate. **(A)** To determine neurite outgrowth cells were treated without (−) and with 100 nM crotamiton (crota), piceid or honokiol (hono) for 24 h. Neurite outgrowth was determined from 100 cells per genotype and condition for each experiment. Values show means ± SD from three independent experiments. **(B)** Cell death was measured without (untr) or with 10 µM hydrogen peroxide (H_2O_2) and treatment with 100 nM crotamiton (H_2O_2+crota), piceid (H_2O_2+piceid) or honokiol (H_2O_2+hono) for 24 h. Cells were stained with calcein and propidium iodide and cell death was measured by counting live and dead cells from three wells per genotype and treatment for each experiment. Experiments were performed independently three times using two to three mice from each genotype per experiment. Values show means ±SD. ** $p < 0.01$ difference compared to untreated, $^{\#}$ $p < 0.05$ difference compared to wild-type (two-way ANOVA with Tukey´s post-hoc test).

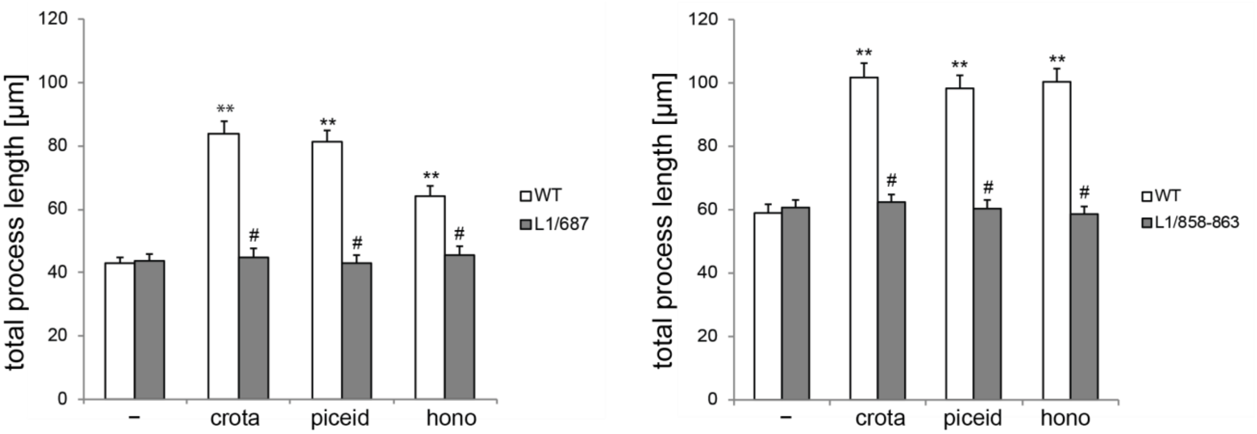

Supplementary Figure S3. Schwann cells from L1/687 and L1/858-868 mice do not respond to stimulation with L1 agonists. Schwann cells were prepared from 6- to 7-day-old male wild-type (WT, white bars) and male L1 mutant littermate (gray bars) mice and seeded on PLL substrate. Process lengths were determined after treatment without (−) and with 100 nM crotamiton (crota), piceid or honokiol (hono) for 24 h. Values from 100 cells per genotype and condition for each experiment were obtained. Experiments were performed independently three times using two to three mice from each genotype per experiment. Values show means ±SD. ** $p < 0.01$ difference to PLL, # $p < 0.05$ difference to wild-type (two-way ANOVA with Tukey´s post-hoc test).
